# Pathogenesis of Chronic Hyperglycemia: From Reductive Stress to Oxidative Stress

**DOI:** 10.1155/2014/137919

**Published:** 2014-06-16

**Authors:** Liang-Jun Yan

**Affiliations:** Department of Pharmaceutical Sciences, UNT System College of Pharmacy, University of North Texas Health Science Center, 3500 Camp Bowie Boulevard, RES-314E, Fort Worth, TX 76107, USA

## Abstract

Chronic overnutrition creates chronic hyperglycemia that can gradually induce insulin resistance and insulin secretion impairment. These disorders, if not intervened, will eventually be followed by appearance of frank diabetes. The mechanisms of this chronic pathogenic process are complex but have been suggested to involve production of reactive oxygen species (ROS) and oxidative stress. In this review, I highlight evidence that reductive stress imposed by overflux of NADH through the mitochondrial electron transport chain is the source of oxidative stress, which is based on establishments that more NADH recycling by mitochondrial complex I leads to more electron leakage and thus more ROS production. The elevated levels of both NADH and ROS can inhibit and inactivate glyceraldehyde 3-phosphate dehydrogenase (GAPDH), respectively, resulting in blockage of the glycolytic pathway and accumulation of glycerol 3-phospate and its prior metabolites along the pathway. This accumulation then initiates all those alternative glucose metabolic pathways such as the polyol pathway and the advanced glycation pathways that otherwise are minor and insignificant under euglycemic conditions. Importantly, all these alternative pathways lead to ROS production, thus aggravating cellular oxidative stress. Therefore, reductive stress followed by oxidative stress comprises a major mechanism of hyperglycemia-induced metabolic syndrome.

## 1. Introduction

Type 2 diabetes is generally an overnutritional disease [1–3]. It is caused by insulin resistance and insulin secretion impairment induced gradually and mainly by high blood glucose in conjunction with other factors such as obesity, aging, genetic predisposition, and physical inactivity [4–9]. Persistent overnutrition creates a steady level of high blood glucose that is toxic to macrovascular and microvascular systems [10–12], an effect known as glucotoxicity [13–17]. While oxidative stress is thought to contribute to the pathogenesis of glucotoxicity during the development of diabetes and diabetic complications [18–26], reductive stress due to excess NADH [27–33] generated by high blood glucose has attracted less attention. In this review, by following the mechanisms of NADH production and recycling, I highlight evidence that reductive stress followed by oxidative stress comprises the fundamental pathogenic mechanisms of chronic hyperglycemia in the development of diabetes and diabetic complications.

## 2. Euglycemia

A normal level of blood glucose below 100 mg/dL is tightly maintained, regulated, and achieved by rate of glucose uptake by all tissues and rate of glucose synthesis by the liver [34] and to a less magnitude by the kidney [35]. Approximately, 75% of the body's total glucose is consumed by insulin-insensitive tissues including the brain, red blood cells, the liver, and the gut, while the rest is consumed by insulin-sensitive tissues including muscle [36]. Postprandially, a rapid increase in blood glucose content stimulates insulin secretion, resulting in a temporary increase in blood insulin concentration known as hyperinsulinemia. The increases in blood concentrations of both glucose and insulin coordinately inhibit glucose production by the liver and facilitate glucose uptake by insulin-insensitive tissues [37]. Therefore, euglycemia is strictly maintained, which is highly dependent not only on proper insulin secretion from the *β*-cells upon nutritional stimulation but also on insulin action in the liver and peripheral tissues [37].

## 3. NADH and Reductive Stress

Electrons from aerobic breakdown of glucose are mainly stored in NADH for oxygen reduction and ATP production. Therefore, NADH is a reducing compound and an excessive amount of it can cause reductive stress [30, 32, 38–40]. Overproduction of NADH or lack of NAD^+^ can induce the accumulation of NADH, leading to imbalance between NADH and NAD^+^ and creating a condition known as pseudohypoxia [29, 41–44]. This is a condition under which oxygen cannot be effectively consumed. This would cause metabolic stress or metabolic syndrome as it often occurs in diabetes [44–47]. It should be noted that GSH and NADPH accumulation, tightly linked to NADH metabolism [48], can also induce reductive stress [39, 49–54]. As mitochondrial complex I is the major enzyme responsible for NADH recycling, impairment of complex I function can thus induce NADH accumulation and reductive stress [55] that could be linked to inhibition of insulin release by *β*-cells [56, 57].

## 4. Hyperglycemia, Elevated Levels of NADH, and Mitochondrial Electron Pressure

**Figure 1:**
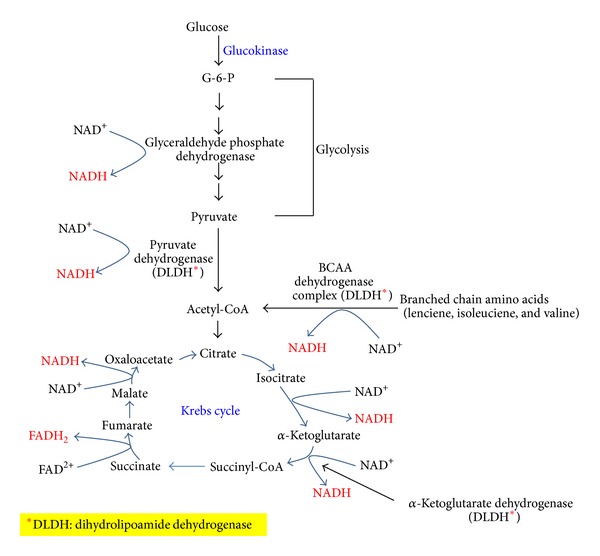
The conventional pathways that generate NADH by breaking down glucose via glycolysis and the Krebs cycle. The enzymes involved in NADH/NAD^+^ recycling are shown. ∗DLDH stands for dihydrolipoamide dehydrogenase and is the component in each given enzyme complex that actually makes NADH from NAD^+^ [191].

The glycolytic pathway breaks down nearly 80%–90% of the body's glucose, while the pentose phosphate pathway consumes the remaining 10%–20% under physiological condition [58, 59]. Under hyperglycemic condition, more glucose will flux through the glycolytic pathway that produces more pyruvate and acetyl-CoA, leading to more NADH production. As NADH is an electron carrier, excess amount of it will cause an electron pressure on the mitochondrial electron transport chain [40, 60–62]. This is particularly true for hepatocytes and pancreatic *β*-cells in that glucokinase (hexokinase D) is a supply-driven enzyme [63], and this enzyme is not inhibited by glucose-6-phosphate (G6P) [64–66]. Therefore, the more glucose the more G6P produced that will be broken down through glycolysis and Krebs cycle, leading to more NADH production.  Figure 1 shows the major conventional pathways that can generate more NADH when glucokinase is used to phosphorylate glucose for glucose breakdown in tissues such as pancreas and liver [67–70].

## 5. NADH-Imposed Electron Pressure and Mitochondrial Superoxide Production

**Figure 2:**
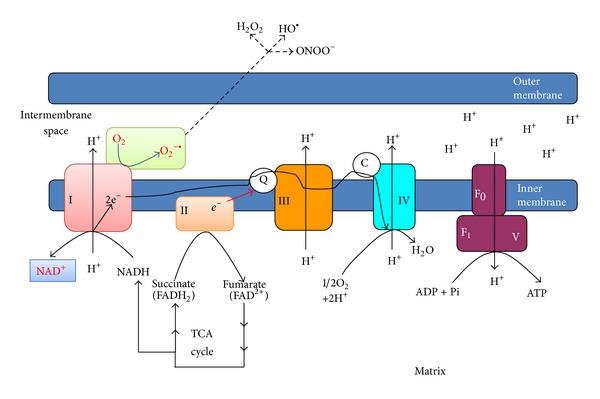
NADH oxidation by complex I in the electron transport chain. Electrons from NADH are transported via CoQ and cytochrome c to molecular oxygen. This process involves proton pumping that is tightly linked to superoxide production. ATP synthesis by complex V driven by the proton gradient is also shown.

The electron pressure induced by overproduced NADH will place a heavy burden on mitochondrial complex I that is the major site for NADH recycling (Figure 2). Under this condition, complex I will respond within its capacity to oxidize more NADH to NAD^+^, in an attempt to ameliorate the pseudohypoxic condition. An inherent nature of NADH flux through complex I is that more superoxide will also be made when more NADH is oxidized by complex I as this complex is also involved in proton pumping [71–73], leading to a proportional increase in electron leakage that will partially reduce oxygen to yield superoxide [71, 74–77]. This scenario could get worse under pseudohypoxic conditions as less NAD^+^ is available for transporting electrons to oxygen [55], leaving more oxygen available for partial reduction by the leaked electrons from complex I and complex III, the latter being also involved in proton pumping [78–80]. It should be noted that complex II and dihydrolipoamide dehydrogenase could also produce superoxide [81–83].

## 6. Superoxide and Oxidative Stress

Superoxide is the precursor of all reactive oxygen species that at elevated levels can cause oxidative stress [84, 85]. As has been established, superoxide can be converted to hydrogen peroxide by superoxide dismutase; hydrogen peroxide can then be converted to form hydroxyl radical by metal ions [84, 86, 87]. In the meantime, superoxide can also react with nitric oxide to produce peroxynitrite (ONOO^−^) [88, 89]. All these reactive species can cause oxidation of proteins, lipids, and DNA [90]. Consequently, an oxidative stress condition has fully developed due to a high level of NADH, achieving the transition from reductive stress to oxidative stress [43, 91–93]. Therefore, reductive stress is not the reverse of oxidative stress; it actually leads to oxidative stress [94, 95].

## 7. Inhibition of Glyceraldehyde 3-Phosphate Dehydrogenase and Alternative Glucose Metabolic Pathways

**Figure 3:**
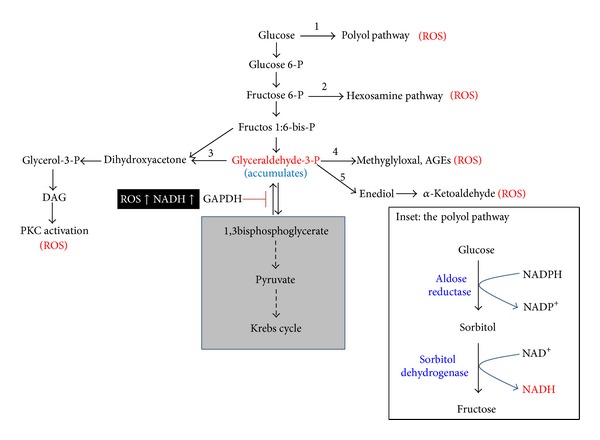
The branch-off pathways that are activated to dispose excess glucose when glyceraldehyde 3-phosphate dehydrogenase (GAPDH) is inactivated by ROS. These five alternative pathways [21, 115], in addition to the electron transport chain shown in Figure 2, are linked to ROS production, thus further exacerbating oxidative stress. Inset shows the polyol pathway. Pathways in the grey area would no longer efficiently break down glucose when GAPDH is inactivated by posttranslational modifications.

As has been discussed above, an oversupply of NADH can lead to overproduction of mitochondrial superoxide and other forms of ROS. These ROS can then impair the activity of glyceraldehyde 3-phosphate dehydrogenase (GAPDH) [22, 96] that is very sensitive to oxidative modifications [21, 97–103] due to a redox-sensitive cysteine residue at its active center [104, 105]. Additionally, high level of NADH would also inhibit GAPDH activity [106]. Such impairments would collectively decrease the efficiency of glucose metabolism via glycolysis and Krebs cycle, inducing accumulation of glyceraldehyde 3-phosphate (G3P). Therefore, all the intermediate products above and including G3P will have to be disposed by pathways that branch off the glycolytic pathways (Figure 3) [107, 108].

## 8. The Branching-Off Pathways and Oxidative Stress

There have been five pathways [21] that can branch off the glycolytic pathway under chronic hyperglycemic conditions (Figure 3). These pathways are minor and insignificant in glucose metabolism under normoglycemic conditions, but can become major pathways to flux high level glucose. As will be discussed below, all the five pathways have been linked to ROS production, oxidative stress, and the pathogenesis of diabetes and diabetic complications [21, 109–115].

### 8.1. The Polyol Pathway

When blood glucose level is high, cellular metabolic pathways change, which usually leads to deleterious effects [5]. A major pathway that is activated in response to hyperglycemia is the polyol pathway [44, 116–118], in which glucose is reduced by aldose reductase to form sorbitol, and the formed sorbitol is then converted to fructose by sorbitol dehydrogenase. This pathway, as shown in Figure 3 (Inset), converts NADPH to NADH using two step reactions and leads to redox imbalance between NADH and NAD^+^. As the ratio of NAD^+^/NADH decreases due to an increase in NADH content, reductive stress can ensue. Because aldose reductase has a very high Km for glucose [119], it can only be activated by a high level of glucose. Hence, this enzyme could also be considered as a supply-driven enzyme [120, 121]. Under hyperglycemic conditions, the polyol pathway has been estimated to utilize more than 30% of the body's glucose [101]. Therefore, this pathway can also contribute significantly to reductive stress [32, 119] and has been thought to play an important role in the pathogenesis of diabetic complications [122–125].

Additionally, in the first reaction of the polyol pathway (Figure 3 inset), NADPH is consumed and, when NADPH level goes lower, so does reduced form of glutathione (GSH). This is because glutathione reductase needs NADPH to regenerate GSH from GSSG (oxidized form of glutathione) [126]. As GSH level goes lower, cellular antioxidant capacity can be compromised, resulting in elevated levels of reactive oxygen species that can attack macromolecules and induce oxidative damage [126]. Therefore, the polyol pathway is also a source of oxidative stress [127–129]. It should also be pointed out that activation of the polyol pathway in return will further decrease glucose consumption by the glycolytic pathway as sorbitol dehydrogenase competes with GAPDH for NAD^+^ [130, 131]. Moreover, as nitric oxide synthase also uses NADPH as a cofactor, a lowered level of NADPH can lead to a decrease in nitric oxide production, thereby facilitating vasoconstriction and platelet aggregation [132].

### 8.2. The Hexosamine Pathway

This pathway branches off from fructose 6-phosphate in the glycolytic pathway. Fructose 6-phosphate is the substrate of the enzyme glutamine-fructose 6-P amidotransferase (GFAT), which is the rate-limiting enzyme for this pathway. GFAT makes glucosamine 6-P from fructose 6-P and the former is further converted to UDP-N-acetylglucosamine, which is the substrate for specific O-GlcNAc transferase that catalyzes posttranslational modifications of proteins via O-GlcNAc on serine and threonine residues [133–135]. Increased glucose flux through this pathway has been shown to be involved in ROS generation and oxidative stress [136–138] and has been implicated in diabetic complications [139–142].

### 8.3. The Protein Kinase C Activation Pathway

Fructose 1:6-bisphosphate can break down to form dihydroxyacetone phosphate and glyceraldehyde 3-phosphate with the former being readily isomerized to glyceraldehyde 3-phosphate under the action of triose phosphate isomerase. Accumulation of glyceraldehyde 3-phosphate can increase the synthesis of diacylglycerol that is an activator of protein kinase C (PKC). PKC activation is known to be involved in elevating the content of TGF-*β*-1, endothelin-1, NF-*κ*B, and vascular endothelial growth factor [22, 143, 144] and is also known to induce ROS production by NADPH oxidase that catalyzes one electron reduction of molecular oxygen to form superoxide [145–147]. Mechanistically, it has been established that PKC activates NADPH oxidase by phosphorylating the p47^phox^ subunit, triggering the translocation of this subunit from cytosol to membrane whereby it assembles with other components to form an active NADPH oxidase that is capable of making superoxide from oxygen [148, 149]. PKC activation can also induce insulin resistance by inhibiting Akt-dependent nitric oxide synthase function [150].

### 8.4. Advanced Glycation End Products (AGEs)

In addition to the polyol pathway, this pathway has also been thought to be a major mechanism of oxidative stress under hyperglycemic condition [151, 152]. High level of glucose can induce formation of methylglyoxal from glyceraldehyde 3-phosphate when GAPDH function is impaired. Methylglyoxal can modify proteins via glycation of amino groups on proteins [153, 154]. One of the major products is glycated hemoglobin (HbA1c) that has been used as a biomarker for diabetes [155, 156]. Therefore, this nonenzymatic process can greatly impair protein function. Moreover, this glycation pathway is known to liberate ROS [157, 158] and upregulate the expression of cell surface receptor for AGEs, leading to activation of the NF-*κ*B signaling pathway and chronic inflammation [159–161].

### 8.5. The Glyceraldehyde Autoxidation Pathway

This pathway also branches off from glyceraldehyde 3-phosphate in the glycolytic pathway. Glyceraldehyde 3-phosphate is formed from fructose 1:6-bisphospate by the enzyme aldose. Under certain conditions, glyceraldehyde 3-phosphate can undergo autoxidation [162], a process that can generate hydrogen peroxide and *α*-ketoaldehydes in diabetes mellitus [21, 163].

## 9. Oxidative Stress, Diabetes, and Diabetic Complications

As discussed above, all the sources of ROS and oxidative stress can be traced back to high blood glucose and NADH overproduction. Therefore, chronic hyperglycemia would inevitably cause chronic reductive stress that leads to oxidative stress. As ROS production is a common feature of the above described pathways [119, 164], chronic oxidative stress certainly plays a central role in the development of diabetes and diabetic complications [22, 165, 166]. Indeed, it has been reported that ROS can induce insulin resistance [74, 167], impair insulin synthesis [168], and impair beta cell insulin secretion [97, 169]. Additionally, oxidative stress biomarkers have been shown to be increased in individuals who exhibit insulin resistance [170–173] or insulin secretion impairment [174–177], indicating a positive correlation between oxidative stress and insulin resistance and insulin secretion impairment. Moreover, numerous studies have also established that ROS are involved in the etiology of diabetic complications including retinopathy, neuropathy, cardiomyopathy, and nephropathy [123, 178–182]. Given that oxidative stress originates from NADH-imposed reductive stress [31, 183], attenuating hyperglycemia-triggered reductive stress may provide potential therapeutic approaches for preventing the development of diabetes and diabetic complications.

## 10. Conclusion

**Figure 4:**
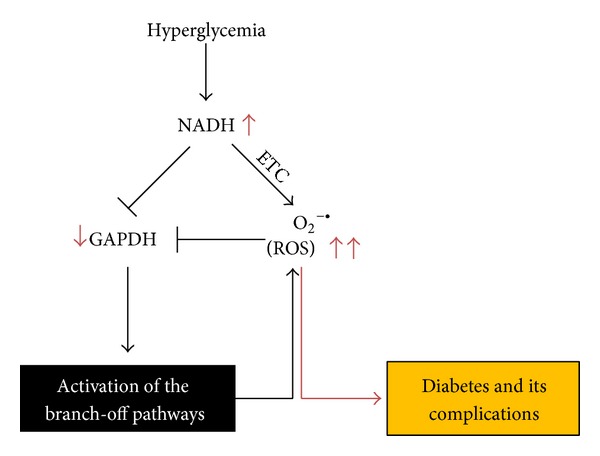
Hyperglycemia induces overproduction of NADH and mitochondrial ROS that inhibit GAPDH activity. This inhibition then activates the alternative glucose metabolic pathways, which further produce ROS involved in glucotoxicity that is responsible for the development of diabetes and diabetic complications. ETC: electron transport chain.

Persistent high blood glucose is highly toxic [16, 112]. It not only induces insulin resistance but also impairs insulin secretion by pancreatic *β*-cells [184]. Over time, hyperglycemia will produce detrimental effects on macrovascular and microvascular systems [185, 186].  Figure 4 summarizes schematically the pathways discussed in this review and their pathogenic roles in chronic hyperglycemia via NADH, ROS, and oxidative stress. As hyperglycemia results in excessive production of acetyl-CoA that feeds into the Krebs cycle, making excess NADH, mitochondrial electron transport chain is thus under heavy electron pressure [40, 60, 61]. Therefore, oxidation of the overproduced NADH by mitochondria will inevitably lead to production of more superoxide and hence more ROS [187, 188], which can in turn attack and inactivate GAPDH. This would trigger the accumulation of glycolytic metabolites upstream of glyceraldehyde 3-phosphate and activate the alternative glucose disposal pathways that all are linked to ROS production and hence increase the magnitude of oxidative stress [21, 189, 190]. Therefore, reductive stress followed by oxidative stress could serve as the major mechanism of glucotoxicity under chronic hyperglycemic conditions. An increase in NADH oxidation by mitochondria without an accompanying increase in ROS production may be a potential therapeutic approach for diabetes and diabetic complications.
